# Analysis of plasma exosomal differential proteins and bioinformatics in intrahepatic cholestasis of pregnancy

**DOI:** 10.3389/fgwh.2026.1751936

**Published:** 2026-04-10

**Authors:** Yuxuan Jiang, Lei Nie, Zhiyuan Liang, Li He, Liming Shen, Danqing Zhao

**Affiliations:** 1 Department of Obstetrics and Gynecology, Affiliated Hospital of Guizhou Medical University, Guiyang, China; 2 Department of Obstetrics and Gynecology, Chengdu Women’s and Children’s Central Hospital, Sichuan, China; 3 College of Life Science and Oceanography, Shenzhen University, Shenzhen, China

**Keywords:** biomarkers, complement and coagulation cascade, exosomes, intrahepatic cholestasis of pregnancy, swath

## Abstract

**Introduction:**

Intrahepatic cholestasis of pregnancy (ICP) is a gestational liver disorder characterized by maternal pruritus and elevated serum bile acids, which is associated with an increased risk of adverse fetal outcomes. The identification of stage-specific diagnostic markers of ICP is crucial for timely diagnosis and stratified intervention.

**Methods:**

This study aimed to identify differentially expressed proteins (DEPs) from patients with ICP at different stages of severity using sequential window acquisition of all theoretical fragment ions (SWATH) proteomics, and to provide preliminary insights into the underlying pathology of the disease. We performed quantitative proteomic profiling of both total exosomes and placenta-derived exosomes isolated from the plasma samples of 35 pregnant women, including 10 with moderate ICP, 10 with severe ICP and 15 healthy controls.

**Results:**

SWATH proteomics identified 109 and 46 DEPs in the ICP/CTR comparison for of total exosomes (T-EXO) and placenta-derived exosomes (P-EXO), respectively. Bioinformatics analysis revealed that these proteins are involved in complement activation, blood coagulation and stress responses in pregnant women with ICP. Through the screening of hub proteins, we identified HRG, VWF and PIGR as three key proteins, which were verified by Western blotting.

**Discussion:**

We hypothesize that elevated maternal total bile acids (TBA) may induce a fetal stress response, leading to the release of procoagulant factors into the maternal bloodstream. Furthermore, PIGR was consistently upregulated in both plasma and placental exosomes, suggesting its potential involvement in the pathogenesis of ICP.

## 1 Introduction

Intrahepatic cholestasis of pregnancy (ICP) is a typical reversible cholestasis that occurs in the third trimester of pregnancy (1, 2). Elevated maternal serum bile acids exert direct toxic effects on multiple fetal organ systems, including the heart, lungs, liver, and nervous system, thereby increasing the risk of adverse pregnancy outcomes such as preterm birth, fetal distress and intracranial hemorrhage in the newborn (3, 4). Studies have shown that there is a positive correlation between the incidence of adverse pregnancy outcomes and the concentrations of bile acids, and the perinatal mortality rate is approximately 6–10 times higher than that of a normal pregnancy (4–8). Beyond its fetal implications, ICP may also have short- or long-term consequences for maternal health. Affected women are at increased risk of developing preeclampsia, jaundice, or steatosis. Later in life, there is also an increased risk of immune-mediated diseases, cardiovascular disease and even advanced liver and bile cancers (9, 10).

The pathogenesis of ICP is multifactorial, and its diagnosis remains one of exclusion, and its diagnosis and classification are usually based on the concentration of total bile acid (TBA) and related symptoms, such as the presence of pruritus. However, this approach has limitations in terms of both sensitivity and specificity (11, 12). The risk of stillbirth in some clinically asymptomatic ICP patients is similar to, or may even be higher than, that of symptomatic ICP patients. In addition, there are obvious limitations to measuring only TBA concentration, such that the results can be easily confused with other liver diseases. Therefore, the selection of blood diagnostic indicators and the search for new specific biomarkers are important for the diagnosis and classification of ICP (13, 14).

Exosomes (EXOs) are vesicles tasked with intercellular signaling that regulate different biological processes in target tissues and are involved in the onset and progression of several diseases (13–17). Recent studies have shown that placental cells communicate with maternal tissues through extracellular vehicles (EVs), thereby regulating their biological functions (18). Placenta-derived exosomes (P-EXO) is a collective term for micro vesicles released by placental cells, characterized by the specific expression of placental alkaline phosphatase (PLAP), which serves as a reliable marker for their identification and quantification (19). It has been shown that P-EXO is progressively released into maternal peripheral blood from the 6th week of gestation and can be measured from it by the 8th week of gestation, with a positive correlation between its amount and gestational age (20). Clearly, with exosomes, we can trace the source of protein changes and explore protein differences in ICP patients.

In view of the important functions and characteristics of exosomes, blood exosomes, especially P-EXO, have attracted extensive attention. There have been some reports on the research of diagnostic markers and disease mechanisms of pregnancy-related diseases (21–28). At present, the research on ICP exosomes mainly involves the study of exosomal microRNA (29–32). Proteins are the bearers of life activities, and their changes can more directly reflect the development of diseases. Most of the disease markers and most of the drug targets are proteins. To the best of our knowledge, there are only two proteomics studies on blood exosomes of ICP (33, 34) and the proteomic study on P-EXO of ICP has not been reported. In this study, peripheral blood exosomes (i.e., total exosomes) and P-EXO were extracted from ICP patients and controls. SWATH (sequential window acquisition of all theoretical fragments)-based proteomic technology was used to compare and analyze the differentially expressed proteins (DEPs) of T-EXO and P-EXO between the two groups, so as to explore the disease mechanism and screen potential disease biomarkers.

## 2 Material and methods

### 2.1 Clinical information and sample collection

**Figure 1:**
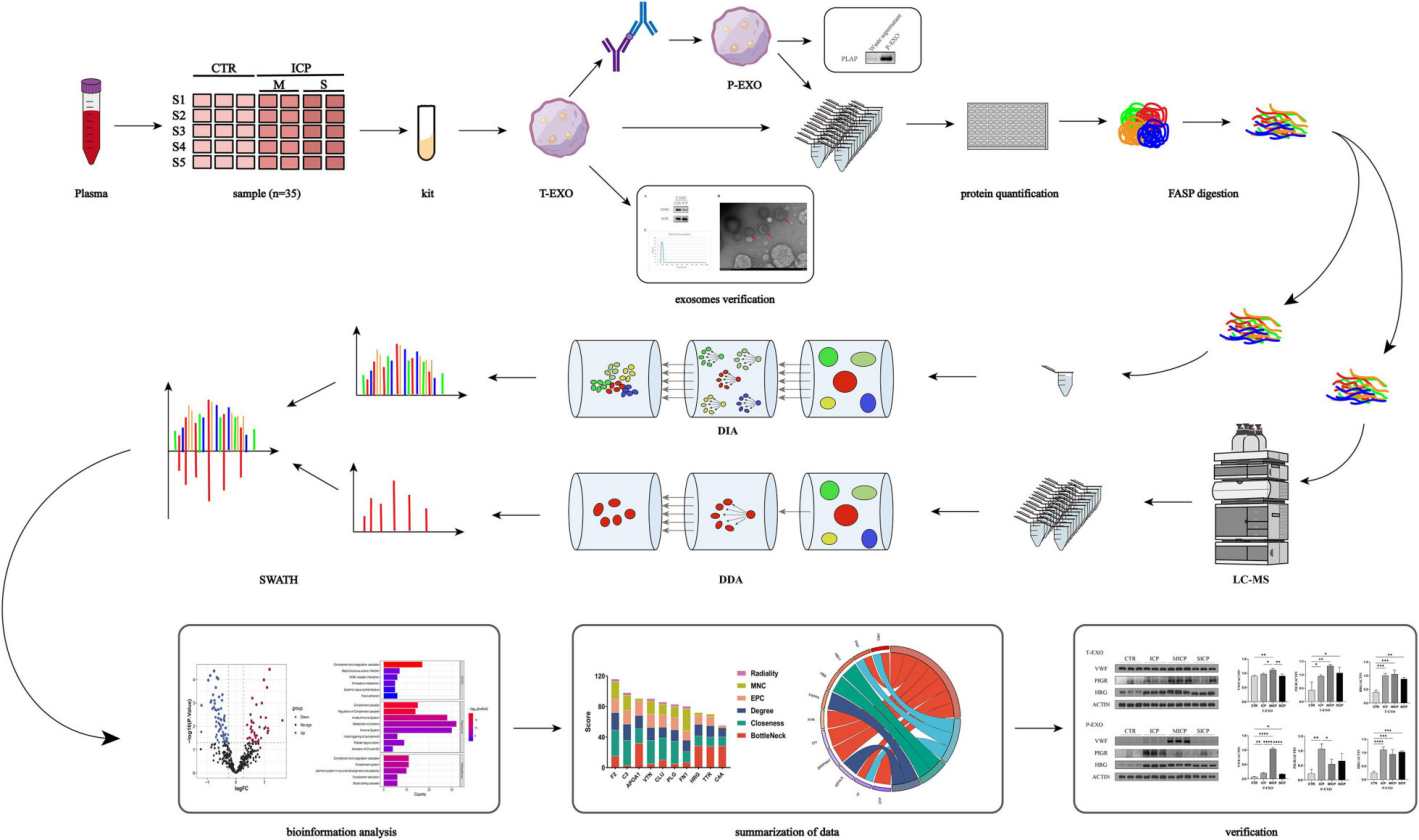
Experimental flow chart.

An overview of the workflow used in this study is shown in Figure 1. The study population was recruited from the Affiliated Hospital of Guizhou Medical University (Guiyang, China). Fasting peripheral blood samples were collected in the morning from pregnant women during the third trimester of pregnancy using ethylenediaminetetraacetic acid (EDTA) anticoagulation tubes (Becton Dickinson Inc., Plymouth, UK). The degree of disease progression was grouped according to the 2015 guidelines for the diagnosis and treatment of ICP recommended by the Obstetrics and Gynecology Section of the Chinese Medical Association (1). Twenty pregnant women were diagnosed with ICP (ICP group), and another 15 pregnant women matched for age, gestational stage, and gestational week served as the healthy control (CTR group). Total bile acid (TBA) concentrations were measured in fasting serum samples by the enzymatic cycling method. The normal reference range for TBA was <10 μmol/L. Based on TBA concentrations, patients with ICP were subdivided into mild ICP (MICP group, TBA 10–39 μmol/L) and severe ICP (SICP group, TBA ≥40 μmol/L). The purpose and content of this study were approved by the Ethics Committee of Guizhou Medical University, and informed consent was obtained from all participants.

### 2.2 Extraction and identification of exosomes

Plasma exosomes were extracted using the Minute^™^ efficient exogenic deposition kit (EI-027, Invent Biotech, USA). The resulting exosome precipitates were redissolved in Dulbecco's Phosphate Buffered Solution (DPBS). The samples were divided into three parts. One part was added to RIPA lysate as total exosome (T-EXO) assay sample, one part was used for exosome identification, and the remaining part was combined with placental alkaline phosphatase polyclonal anti-body (PLAP, PA5-112357, Invitrogen, USA), 3% bovine serum albumin (BSA) and agarose purified resin to obtain P-EXO (22).

Exosome isolation and characterization were performed following the Minimal Information for Studies of Extracellular Vesicles (MISEV) 2023 guidelines (35). The initial protein concentration of exosomes was determined using the BCA kit (CAS No. KGP903, KeyGEN Bio-TECH, China). Samples were diluted with DPBS as needed. Exosome identity and purity were confirmed by Western blot analysis, transmission electron microscopy (TEM) (HT7700, Hitachi, Japan) and nanoparticle tracking analysis (NTA) (ZetaView, Particle Metrix, Germany). Western blot analysis was performed with reference to the previous study (22). The sample loading amount of each sample was 20 µg, protein samples were separated by 12% SDS-PAGE (sodium dodecyl sulfate-polyacrylamide gel electrophoresis), and then transferred to polyvinylidene fluoride (PVDF) membranes, and blocked for 2 h with 5% skimmed dry milk in Tris-buffer saline (TBS: 100 mM Tris, 1.5 mM NaCl, pH 7.6), and washed with TBS containing 0.4% tween 20 (v/v) TBS (TBST), and incubated with anti-CD63 antibody (Abcam, Cambridge, UK, No: ab134045; 1:10,000 dilution), anti-ALIX antibody (Abcam, No: ab275377; 1:1,000 dilution) overnight with, respectively. After washing with TBST, the membranes were incubated with diluted horseradish peroxidase (HRP)-conjugated secondary antibody (Abmart Inc, Shanghai, China). The membranes were then washed with TBST and visualized with an enhanced chemiluminescence (ECL) kit (FDbio Science Biotech co, Ltd, Hangzhou, China). Immunoreactive signals were detected using a Kodak Image Station 4000MM imaging system (Carestream Health Inc., Rochester, NY, USA). For P-EXO, the Western blot analysis was carried out as described above, and anti-PLAP antibody (Abcam, No: ab133602; 1:20,000 dilution) was used as the primary antibody for detection.

For TEM analysis, the diluted exosome solution was taken and dropped onto the front side of a carbon support membrane (copper mesh), left to stand for 5–10 min, and the remaining liquid was removed with filter paper. Subsequently, 7.5 µL of 1% uranyl acetate staining solution was applied to the membrane and stained in the dark for 1–2 min. For NTA analysis, exosomes were diluted to the appropriate concentration and loaded into a colorimetric dish for analysis using a particle size analyzer (ZetaView, Particle Metrix, Germany).

### 2.3 Information dependent acquisition (IDA) mode to create reference spectral library for SWATH

The SWATH-mass spectrum (MS) analysis requires a reference spectrum library. The samples used for quality control (QC) were combined samples containing all the samples to be tested, so we extracted 20 μg of peptide from each sample to be tested to form the QC samples. Liquid chromatography analysis was performed using high-pH reverse-phase (RP) chromatography (Durashell, C18, 250 mm × 4.6 mm, 5 μm; Bonna-Agela Technologies Inc., Wilmington, DE, US), with 1 min intervals to collect 24 fractions of peptides. Data were collected in IDA mode in a Triple TOF 6600 (AB SCIEX, Framingham, MA, US) mass spectrometer. The first 40 strongest ions with multiple charged ions were scanned sequentially.

### 2.4 SWATH-MS analysis

The SWATH-MS analysis was performed based on the DIA model, and the manipulations were performed as previously described (36, 37). During the DIA assay, individualized batch scripts for different experiments was controlled the order in which the samples are detected, and information about the samples is captured in an alternating fashion. As for quality control analysis, QC samples were detected every five samples. At the end of the acquisition process, ProteinPilot Version 5.0 (AB SCIEX, Framingham, MA, US) processed the IDA data files by the ParagonTM algorithm, whereas the SWATH data files were processed by PeakView Version 2.0 (AB SCIEX, Framingham, MA, US).

### 2.5 Multivariate statistical analysis of proteomic data and identification of differential expressed proteins (DEPs)

Protein identification required at least one peptide and quantification requires two peptides, and these steps were performed by the software ProteinPilot Version 5.0. Further analysis of the quality control normalized peak intensities was performed using the StatTarget R package (38). Principal components analysis (PCA), partial least squares-discriminant analysis (PLS-DA) and permutation test were performed by using SIMCA-P (version 14.1, Sartorius Stedim Data Analytics AB, Umea, Sweden). DEPs were analyzed by using R package Limma (v3.48.3), 1.2-fold change and 0.83-fold change with an adjust *p*-value <0.05 were used as screening conditions for up-regulated and down-regulated proteins between different groups, respectively.

### 2.6 Bioinformation analysis of differentially expressed proteins

The pathway enrichment analysis of Gene ontology (GO), Kyoto Encyclopedia of Genes and Genomes (KEGG) was performed using the R package clusterprofiler (v4.2.1), whereas protein-protein interaction (PPI) network as well as Reactome and Wiki pathways were analyzed by STRING (https://cn.string-db.org/) and Cytoscape (Version 3.7.1). The volcano plots and cluster heatmaps were drawn using the R packages ggplot2 (v3.3.5) and pheatmap (v1.0.12). The hub proteins were identified using CytoHubba plugin.

### 2.7 Western blot analysis validated important differentially expressed proteins

The expression of important DEPs obtained through pathway analysis and hub protein screening was verified. We also recruited 20 pregnant women with ICP (10 with mild ICP and 10 with severe ICP) and 10 healthy pregnant women matched for age, gestational age, and body mass index (BMI) for this validation step. The primary antibodies involved were: anti-HRG (1:10,000, Proteintech, No: 26252-1-AP), anti-VWF (1:2,000, Proteintech, No: 27186-1-AP) and anti-PIGR (1:2000, Affinity Biosciences, No: DF16099).

### 2.8 Statistical analysis

Statistical analysis was calculated with GraphPad Prism Version 9.0 (GraphPad Software, San Diego, USA). Data were presented as mean ± standard deviation (SD). The non-paired and two-tailed of *t* test (and nonparametric tests) and one-way ANOVA (and nonparametric or mixed) were applied to determine whether a significant (*p* < 0.05).

## 3 Results

### 3.1 Clinical information

The clinical information of the participants recruited for this study is shown in Supplementary Table S1. Compared with CTR group, there were no statistical differences in age, body mass index (BMI), fetal birth weight, and gestational age in the disease group (*p* > 0.05), while the levels of TBA, alanine aminotransferase (ALT) and aspartate aminotransferase (AST) in ICP groups were significantly higher than those in CTR group (*p* < 0.05). There were significant differences in total bilirubin (TBIL), direct bilirubin (DBIL) and high-density lipoprotein cholesterol (HDL-C) between SICP group and CTR group (*p* < 0.05).

### 3.2 Characterization of plasma exosomes

**Figure 2:**
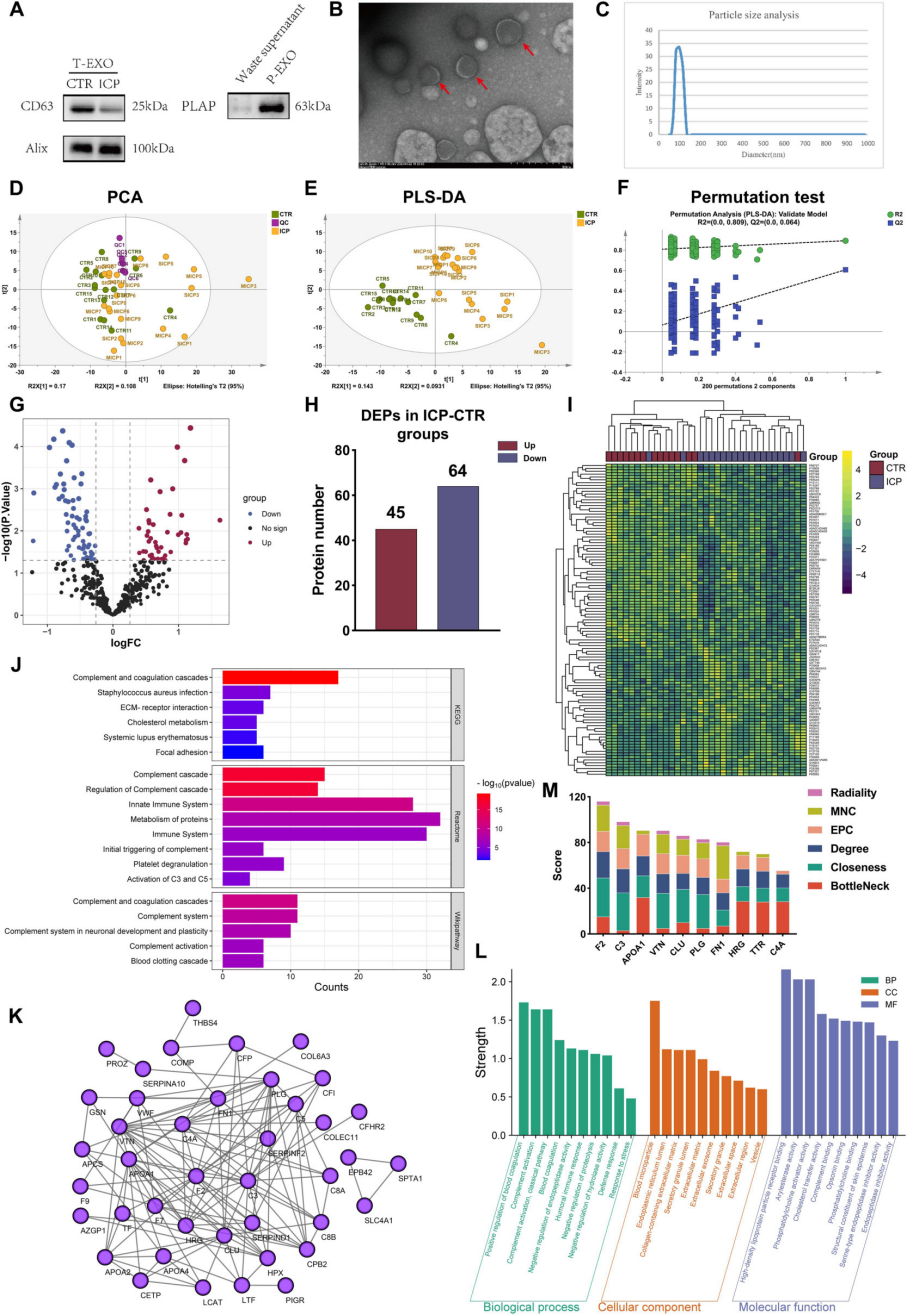
Validation of exosome extraction and screening of differentially expressed proteins (DEPs) in total exosomes (T-EXO). **(A)** Western blot showed the expression of specific proteins (CD63 and Alix) in total exosomes, and PLAP in placenta-derived exosomes. **(B)** Electron microscopy shows that exosomes attached to 230 mesh carbon support film. Bar indicates 500 nm. **(C)** Particle size analysis shows that the size of exosomes is about 100 nm and has a high abundance. **(D)** PCA plot among QC, ICP and CTR group. **(E)** PLS-DA validation plot among QC, ICP and CTR group. **(F)** PLS-DA validation plot (permutation = 200). **(G–I)** Volcano plot of DEPs, the number of up-regulated proteins and down-regulated proteins, and the cluster heatmap of DEPs in the ICP/CTR group. **(J)** The KEGG pathways of DEPs in the ICP/CTR group. **(K)** The PPI network of DEPs in ICP-CTR group. **(L)** The GO analysis of DEPs in ICP/CTR group. **(M)** The hub proteins of DEPs in ICP/CTR group using different algorithms.

We identified the exosomes isolated from plasma in three ways: exosome marker proteins validation, particle size distribution, and electron microscopy detection. By Western blot analysis, we found that ALIX and CD63, which are exosome marker proteins, could be detected in the total exosomes we extracted. Furthermore, PLAP protein showed high expression in extracted P-EXO, which indicated that P-EXO was successfully extracted (Figure 2A). TEM revealed disc-shaped, bilayer membrane-bound vesicles with diameters ranging from 100 to 150 nm. These vesicles exhibited electron-dense outer membranes, moderately electron-dense contents, and a characteristic halo-like appearance (Figure 2B). NTA showed that the particles were mainly distributed in the range of 50–200 nm, with an average value of 100 nm, and there were no abnormal size particles (Figure 2C) (29, 33, 39).

### 3.3 SWATH-based proteomic analysis of the T-EXO in ICP/CTR group

SWATH-MS was applied to identify the proteome of T-EXOs in ICP and CTR groups. A total of 898 proteins were identified and quantified, of which 520 quantified proteins met the quality control requirement after QC correction (Supplementary Table S2). The unsupervised PCA analysis revealed an overlap of T-EXO in the ICP and CTR groups, but QC samples clustered tightly together, confirming the stability and reliability of the data (Figure 2D). Supervised PLS-DA analysis showed a clear trend of separation between the ICP and CTR groups, which were able to be completely differentiated (Figure 2E). 200 permutation tests confirmed that the model was robust with no overfitting (R2 = 0.809, Q2 = 0.064) (Figure 2F). Volcano plot analysis showed that 109 DEPs were identified between the ICP group and CTR group, of which 45 were up-regulated and 64 were down-regulated (Figures 2G,H, Supplementary Table S2). Cluster analysis of the DEPs showed that most samples clustered according to their groups (Figure 2I).

By GO analysis, these DEPs were localized in extracellular space, extracellular exosome, blood microparticle and extracellular region, and were mainly enriched in biological processes such as positive regulation of blood coagulation, complement activation, humoral immune response, negative regulation of proteolysis, negative regulation of hydrolase activity, defense response, response to stress (Figure 2L, Supplementary Table S3). Pathway enrichment analysis revealed that they were predominantly enriched in the complement and coagulation cascades, cholesterol metabolism, systemic lupus erythematosus, and focal adhesion (Figure 2J, Supplementary Table S4). The relationships of the proteins involved in the above pathways were all demonstrated by the PPI network. These proteins were enriched in multiple pathways, indicating that they might be important regulatory proteins (Figure 2K). Based on these findings, we followed up with a hub protein screening, 10 proteins, including F2, C3, APOA1, VTN, CLU, PLG, FN1, HRG, TTR, and C4A were assigned high score in various algorithms including Radiality, M NC (Maximal Neighborhood Component) and EPC (Edge Percolated Component) (Figure 2M). Of these, except for TTR, which was not enriched in PPI network, the remaining 9 proteins were closely related to other proteins in PPI network (Figure 2K). The results suggest that they have the potential to be used as diagnostic biomarkers.

### 3.4 Analysis of DEPs of T-EXO in mild, severe ICP and control groups

Using the same analytical method, we analyzed the MICP, SICP, and CTR groups. Unsupervised PCA analysis showed that the QC samples were clustered, however, there was a varying degree of overlap between the samples of each group. We followed up with supervised PLS-DA analysis, which showed a more pronounced trend of separation between the groups, and the 200 permutation tests indicated that the model was not overfitted (Supplementary Figure S1A–C). Screening of DEPs showed 22 up-regulated proteins and 59 down-regulated proteins in MICP/CTR group; 34 up-regulated proteins and 49 down-regulated proteins in SICP/CTR group; 13 up-regulated proteins and 5 down-regulated proteins in SICP/MICP group (Supplementary Figure S1D–G and Table S5).

In addition, we also explored the common DEPs from different groups, from the Venn plot of T-EXO, we found that the protein PIGR (P01833) was identified as a DEP in all groups, and the expression of this protein showed an increasing trend from the onset of the disease to the progression of the disease to the severity, suggesting that this protein may be an important protein for disease progression (Supplementary Figure S1O and Table S6).

Subsequently, we performed bioinformatics analysis on 44 common DEPs in the MICP/CTR and SICP/CTR groups, the results of GO analysis showed that most of them were located in blood microparticle, collagen-containing extracellular matrix and endoplasmic reticulum lumen, involved in biological processes such as blood coagulation, complement activation and humoral immune response, and had molecular functions like serine-type endopeptidase inhibitor activity, endopeptidase inhibitor activity and sulfur compound binding. Pathway analysis showed that these proteins were associated with the abnormality of complement and coagulation cascades, which is highly similar to the results of ICP/CTR analysis, suggesting that the complement and coagulation cascade plays a rather important role in the disease (Supplementary Figure S2A,B and Table S6, S7).

### 3.5 SWATH-based proteomic analysis of the P-EXO in ICP/CTR group

**Figure 3:**
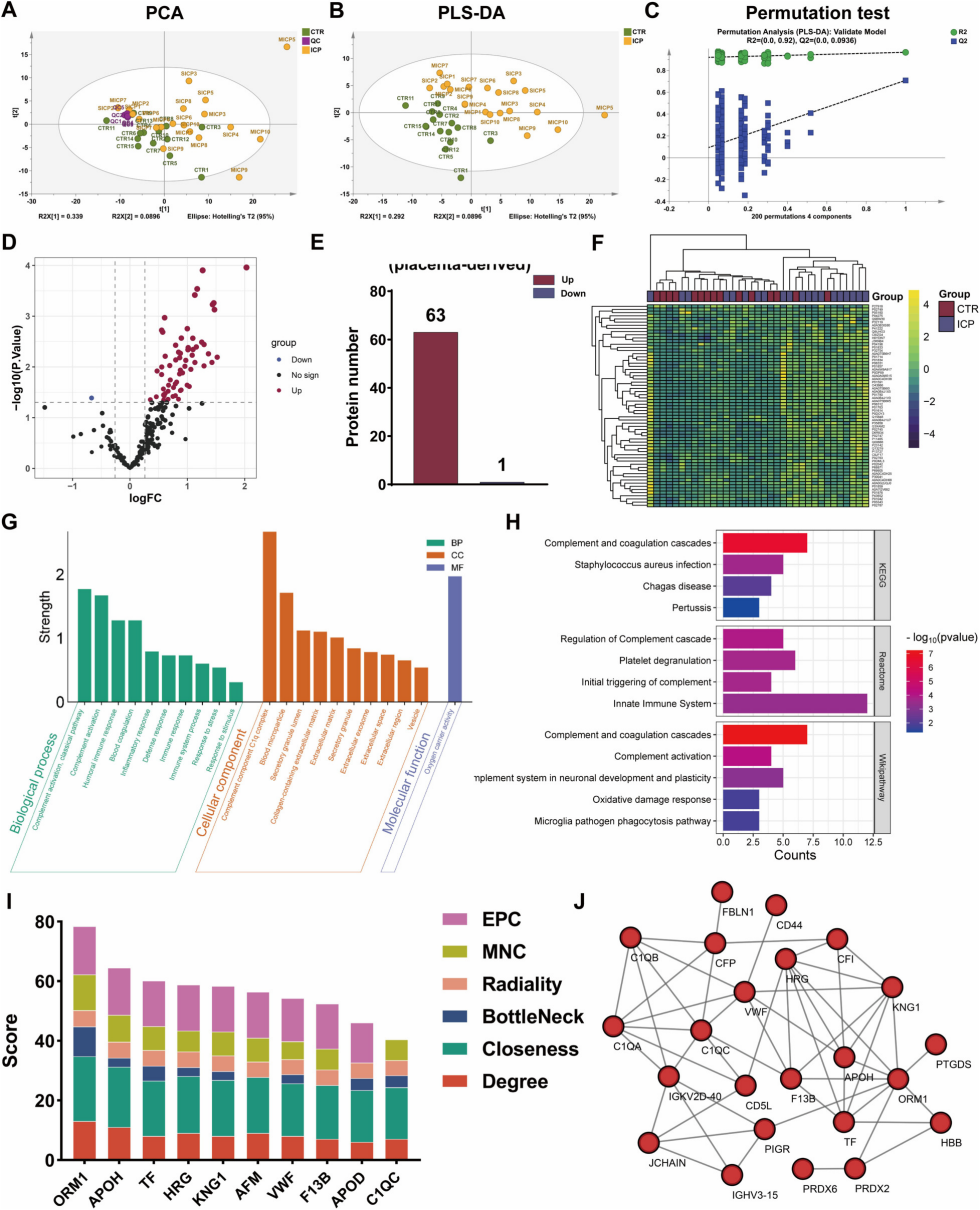
Screening of differentially expressed proteins (DEPs) in placenta-derived exosomes (P-EXO). **(A)** PCA plot among QC, ICP and CTR group. **(B)** PLS-DA validation plot among QC, ICP and CTR group. **(C)** PLS-DA validation plot (permutation = 200). **(D–F)** Volcano plot of DEPs, the number of up-regulated proteins and down-regulated proteins, and the cluster heatmap of DEPs in the ICP/CTR group. **(G)** The GO analysis of DEPs in ICP/CTR group. **(H)** The KEGG pathways of ICP/CTR group DEPs. **(I)** The hub proteins of DEPs in ICP/CTR group using different algorithms. **(J)** The PPI network of differentially expressed proteins in ICP/CTR group.

SWATH-based proteomic analysis was also performed on P-EXO extracted from the ICP and CTR groups. The results showed that a total of 410 quantitative proteins were identified by SWATH-MS analysis, of which 259 proteins met the quality control requirement. In the unsupervised PCA analysis, the CTR and ICP groups showed an overlap, but the QC samples were closely clustered together, indicating the stability and reliability of the data (Figure 3A). The subsequent supervised PLS-DA analysis showed that the CTR group could be distinguished from the MICP and SICP groups, and although there was a tendency for separation between the MICP group and the SICP group, there was overlap (Figure 3B). The 200 permutation tests confirmed that the model was robust and overfitting did not occur (R2 = 0.92, Q2 = 0.0936, Figure 3C).

Volcano plot analysis showed that there were 64 DEPs in the ICP/CTR group, including 63 up-regulated and 1 down-regulated proteins (Figures 3D,E and Supplementary Table S8). Clustering heatmap analysis of these DEPs showed that a small number of samples from the ICP group overlapped with the CTR group, but the majority of samples in the ICP group were distinguished from the CTR group (Figure 3F). GO enrichment analysis of DEPs from P-EXO in ICP/CTR group showed that DEPs were mainly enriched in BPs such as complement activation, humoral immune response, blood coagulation, inflammatory response, defense response, response to stress, response to stimulus (Figure 3G and Supplementary Table S9).

Pathway analysis showed that these DEPs were mainly enriched in complement and coagulation cascades, pertussis, platelet degranulation, and the innate immune system (Figure 3H and Supplementary Table S10). Hub protein screening showed that 10 proteins including ORM1, APOH, TF, HRG, KNG1, AFM, VWF, F13B, APOD, and C1QC were assigned high scores and had the potential to become diagnostic biomarkers of placental origin (Figure 3I). Among them, except for AFM and APOD, which were not enriched in PPI network, the remaining eight proteins were closely related to other proteins in this network (Figure 3J).

### 3.6 Analysis of DEPs of P-EXO in mild, severe ICP and control groups

We also analyzed the P-EXO data after grouping, and the PCA results showed that the mass spectrometer data was in a stable state (QC sample clustering), while the MICP, SICP, and CTR group samples overlapped but showed a separation trend. In the PLS-DA model, the separation trends of the three groups were more pronounced, especially between the CTR group and the MICP and SICP groups. The permutation test indicated that the model was not overfitting (Supplementary Figure S1H–J). Screening of the DEPs in the different groups showed that the MICP/CTR and SICP/MICP groups had only individually up-regulated or individually down-regulated proteins, with 52 up-regulated proteins in the former and 9 down-regulated proteins in the latter. In addition, 41 proteins were identified as DEPs in the SICP/CTR group, of which 36 were up-regulated proteins and 5 were down-regulated proteins (Supplementary Figure S1K–N and Table S11).

Analysis of common DEPs across comparisons revealed that PIGR was consistently up-regulated in all ICP/CTR, MICP/CTR and SICP/CTR groups (Supplementary Figure S1P and Table S12). Bioinformatic analyses of 27 common DEPs in the MICP/CTR and SICP/CTR groups of P-EXO, which have antigen-binding functions and play a role in biological processes such as humoral immune response, complement activation and antigen receptor-mediated signaling pathway. KEGG analysis further showed that they are enriched in pathways such as complement and coagulation cascades, ubiquinone and other terpenoid-quinone biosynthesis, again emphasizing the importance of complement and coagulation pathways in the disease (Supplementary Figure S2C,D and Table S13).

### 3.7 Common differentially expressed proteins indicated complement and coagulation cascades may be affected

**Figure 4:**
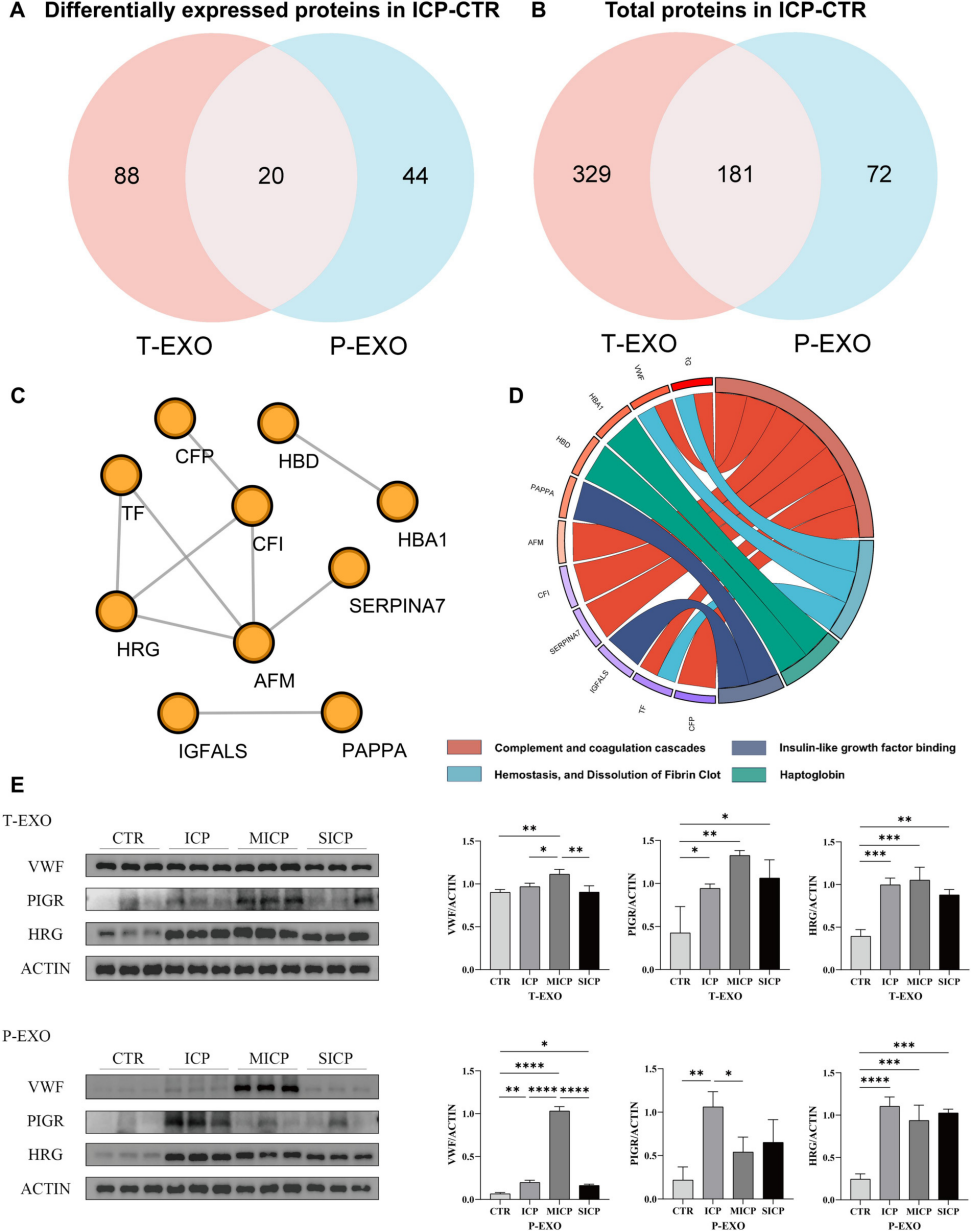
Screening of common DEPs and validation of key proteins from SWATH proteomics. **(A)** Venn plot of DEPs in ICP/CTR between T-EXO and P-EXO. **(B)** Venn plot of total proteins in ICP/CTR between T-EXO and P-EXO. **(C)** The PPI network of common DEPs from ICP/CTR group in T-EXO and P-EXO. **(D)** The GO analysis of common DEPs in ICP/CTR group in T-EXO and P-EXO. **(E)** The representative images and quantification of Western blot analysis bands.

To identify the common biological processes and pathways shared by the proteomes of exosomes derived from different sources, we screened the common DEPs of exosomes from both sources (Figures 4A,B). The results showed that 20 proteins were identified as common DEPs, including HRG, TF, PIGR, CFP, CFI and HBA1. These common DEPs were subsequently used in pathway enrichment analyses, which showed that most of these proteins were enriched in the complement and coagulation cascades, hemostasis, and dissolution of fibrin clot and insulin-like growth factor binding, and pappalysin-1/2 pathways (Figure 4C), indicating that the complement and coagulation systems of ICP patients were affected, similar to our previous analysis of the proteome in exosomes of different sources, warranting further investigation. Protein interaction analysis of these proteins suggests that HRG, AFM, and CFI are of interest for subsequent studies, as they are also important proteins enriched in multiple related pathways simultaneously (Figure 4D).

### 3.8 Western blot analysis and verification

Moreover, we applied Western blot analysis to validate the key DEPs (VWF, PIGR, and HRG) identified in the proteomic analysis. An additional 30 samples, selected by employing the identical criteria used for the initial screening, were analyzed (Supplementary Table S14). The WB results showed that the expression of all three proteins was up-regulated in the ICP group, which is consistent with the proteomics results (Figure 4E).

## 4 Discussion

The results of this study showed that there were 109 DEPs of T-EXO in the ICP/CTR group, of which 45 were up-regulated and 64 were down-regulated. Bioinformatics analysis showed that these DEPs were mainly enriched in biological processes such as complement activation, blood coagulation, humoral immune response, defense response and response to stress. The distribution of cell components was also mostly associated with exosomes, enriched to cellular localizations such as extracellular space, extracellular region and vesicle. The enrichment of KEGG pathway shows that these proteins are related to complement and coagulation cascade, and staphylococcus aureus infection. Protein interactions are complex and close. As an up-regulated protein, VWF is involved in various biological processes such as platelet activation, complement and coagulation cascades, and also plays a certain pivotal role by linking various proteins. Among the screened hub proteins, C3, F2, HGFAC and PLG are mainly involved in the complement and coagulation cascade, proteolytic action, and the down-regulated expression of APOA1 and APOA4 may be the obvious factors of lipid metabolism disorders in ICP patients, and may also be related to the development of atherosclerosis and other cardiovascular diseases (40). HRG can activate platelets and transport heme, and is also involved in angiogenesis and endothelial cell migration.

The DEPs in MICP and SICP compared with the CTR group were associated with abnormal complement and coagulation cascades, which were highly similar to the results of ICP/CTR analysis, suggesting that complement and coagulation cascades play an important role in the disease.

There were 46 DEPs of P-EXO in the ICP/CTR group, of which 45 were up-regulated and 1 was down-regulated. The main biological processes involved in these DEPs are basically the same as those of T-EXO. Among the hub proteins, KNG1, HRG, TF, VWF, and F13B are involved in coagulation, and ORM1 is associated with inflammation.

Through combined analysis, we found that PIGR was up-regulated in all types of T-EXO, and it was also upregulated in the ICP/CTR, MICP/CTR, SICP/CTR groups of P-EXO, and we have reason to believe that PIGR may play an important role in the development of ICP and be associated with disease severity. HRG and VWF appear repeatedly as pivot proteins in this study, and were involved in various biological processes and protein interactions, which will be discussed in detail in this paper.

P-EXO are secreted by placental trophoblast cells into peripheral blood for detection, but no differential expression of PIGR, HRG and VWF was found in our previous placental proteomics studies (41). We considered that on the one hand, the low expression of these may have been masked by other peak proteins in the placenta, so that these differences were not detected. On the other hand, the placenta acts as a barrier between the mother and fetus, and the differential expression of these proteins may be due to the exchange of umbilical cord blood. In this study, we believe that PIGR, HRG and VWF were of significance for further discussion and further study.

When we drew the VENN diagram of the total protein, we found that the total protein of the T-EXO was not exactly the same as that of the P-EXO. This suggests that proteins contained in T-EXO from other cells or tissues can interfere with protein expression in P-EXO. It can also be emphasized that when a protein had statistically significant differences between the T-EXO and the P-EXO, and the differential expression is consistent, we can consider that this protein had important significance in the occurrence and development of the disease, and this differential expression could be preliminarily considered to be caused by the exosomes secreted by the placenta.

It is reported that the activities of alcohol dehydrogenase (ADH), ADH isoenzyme and aldehyde dehydrogenase (ALDH) in serum are indicators of liver cell damage. Some studies have shown that the activities of class I ADH isoenzymes and ALDH in the serum of ICP patients are significantly higher than those of healthy pregnant women and healthy non-pregnant women. The increase of alanine aminotransferase (ALT) in ADH isoenzymes was more significant than that of aspartate aminotransferase (AST), and both are positively correlated with TBA (42, 43). This is consistent with the results of this study, indicating that under different detection methods, the expression of ADH I activity is significantly increased in patients with ICP, which is of great significance for the diagnosis of ICP. Meanwhile, the activity of ADH I in the serum of patients with metastatic liver cancer, autoimmune hepatitis or non-alcoholic fatty liver disease was also significantly higher than that of the healthy control group (44). However, in this study, the protein expression of ADH1A only showed significant differences in the two groups of T-EXO. Therefore, this study further verified and discussed other proteins with more significant differences.

PIGR is a polymerized immunoglobulin receptor that is a first-line antibody against primary infection (45, 46). Previous studies have shown that in viral or bacterial infections, pro-inflammatory cytokines activate the JAK-STAT, NF-κB, and mitogen-activated protein kinase (MAPK) signaling pathways, leading to PIGR overexpression (45, 46), thus linking innate and acquired immunity. As an important inflammatory mediator, PIGR plays an important role in hepatitis B virus infection, chronic liver inflammation, tumor growth, recurrence, and metastasis progression of hepatocellular carcinoma (47–51). Studies have shown that PIGR in extracellular vesicles is almost not expressed in normal hepatocytes, but is highly expressed in hepatocellular carcinoma (HCC) cells, and plays an oncogene role in the occurrence and development of HCC (52–54), which is associated with chemotherapy resistance and/or poor prognosis of cancer. Therefore, PIGR has the possibility to be used as a diagnostic and prognostic marker for HCC.

Of note, reproductive hormones are implicated in the pathogenesis of ICP (55, 56). It usually begins in the last trimester of pregnancy, when estrogen and progesterone concentrations are highest, and resolves when hormone levels return to normal 1 or 2 days after delivery. In addition, twin pregnancies exhibit a higher incidence of ICP (57). Prolactin levels are significantly higher in cholestatic pregnancies than in normal pregnancies (58, 59). Prolactin receptor (PrlR) isoform expression in the kidney is strongly upregulated in cholestasis of pregnancy (60). Interestingly, lactogenic hormones (PRL and cortisol) can upregulate *PIGR* gene expression (61). To our knowledge, no studies of PIGR and pregnancy-related diseases have been reported. In this study, we found that PIGR was present in both T-EXO and P-EXO of various types and showed high expression in pregnant women with ICP, which is consistent with the pathogenic expression trend of PIGR in previous studies, suggesting that it may play a key role in the ICP. It may be associated with inflammatory response, liver damage, high hormone levels in pregnant women with ICP, which is worthy of further study.

HRG is a plasma protein synthesized in the liver that maintains homeostasis of the vascular system by binding to a variety of ligands and interacting with a variety of cells (11, 62). HRG is actively involved in acute inflammation and chronic autoimmune diseases and can also normalize tumor blood vessels and promote anti-tumor immunity (12), thereby reducing tumor growth and metastasis (63, 64). At present, due to the regulatory effect of HRG on blood vessels and coagulation, many studies have discussed HRG as a diagnostic marker for preeclampsia (65–68).

At present, HRG has been shown to have an effect on implantation of fertilized eggs, and homozygous carriers of HRG A1042G SNP and HRG C633T SNP were more likely to have repeated miscarriages, which proved that *HRG* gene polymorphism affects pregnancy outcomes in IVF (67, 69, 70). In one study, serum concentrations of *HRG* were found to be decreased in pregnant women in their third trimester of pregnancy (70).

The establishment of pregnancy requires adequate regulation of angiogenesis, clotting, and immune pathways (71), and defects in a single factor that may interact with other factors may lead to the failure of pregnancy (72). During histological examination of the placenta of pregnant women with ICP, activated maternal platelets were found in the spiral artery lumen (73). In the activated state, platelets release various growth factors, cytokines, and ions stored in the particles. Local increases in zinc ion concentration can induce conformational changes in histidine/proline (His/Pro) -rich domains, thereby promoting their binding to endothelial cells via heparan sulfate and activation of HRG (74, 75). This conclusion can support the increased expression of HRG in this study. We can think that this is a stress effect or negative feedback regulation produced by the fetus during ICP.

VWF, also known as von Willebrand factor, is involved in hemostasis and encourages platelets to adhere to damaged blood vessels. It also acts as a companion for factor Ⅷ, transporting it to the site of injury, stabilizing its heterodimer structure and protecting it from premature clearance by plasma. Plasma VWF is highly expressed in many cardiovascular diseases and neoplasms and can predict the risk of thrombosis (76).

It is well known that pregnancy is a hypercoagulable state, with a significant increase in procoagulant activity in maternal blood, manifested by elevated levels of D-dimer, factors II, VII, VIII and X, fibrinogen and VWF, as well as increased clotting activation markers, especially in the third trimester, and this pre-thrombotic state lasting up to 12 weeks after delivery (77, 78) This study found that the concentration of VWF in the maternal blood of pregnant women with ICP was higher than that of normal pregnant women, which was mutually confirmed by a study in 2006 (79). The concentration of exosome VWF in fetal and neonatal plasma is higher than that in adults (80). In this study, the concentration of VWF in the maternal blood of pregnant women with ICP was found to be increased. It can be assumed that during the progression of ICP in pregnant women, the permeability of the maternal interface to coagulation factors increases, and the fetus introduces a large amount of coagulation factors into the maternal peripheral blood in the form of placental exosomal vesicles, resulting in the pathological hypercoagulability of the mother.

In summary, we conducted proteomic studies on ICP from T-EXO and P-EXO, comprehensively discussed the pathogenesis of ICP, and obtained three proteins that play an important role in the occurrence and development of ICP, and verified them. Western blot strip and gray value analysis showed that the differential expression of the three proteins in different groups was basically consistent with the results of proteomics studies. The above provides a new idea for the diagnosis of ICP.

However, several limitations of this study should be acknowledged. First, there is no standardized method to effectively isolate error-free exosomes. Second, the sample size was small, and all of them came from the same region, so there may be regional differences in the results. Finally, while this study suggests that VWF, HRG and PIGR may play an important role in the occurrence and development of ICP, but the specific causal relationship with the mild and severe disease typing of ICP and TBA changes had not been demonstrated.

## 5 Conclusion

The expression pattern of plasma exosomes in women with intrahepatic cholestasis of pregnancy differs significantly from that in normal pregnant women. The increase of maternal TBA may induce the fetus to produce a protective mechanism of stress, thereby releasing a large number of procoagulant substances into the maternal blood through the maternal-fetal interface, including VWF protein as a partner of FVIII. The procoagulant substances promote the activation of platelets in maternal blood and release a large number of cytokines and ions, among which the increase of zinc ion concentration can indirectly promote the increase of HRG protein expression and make the mother develop a pathological hypercoagulable state. Increased pro-inflammatory factors in third-trimester ICP patients may activate JAK-STAT, NF-κB and MAPK signaling pathways, which may contribute to the overexpression of PIGR.

## Data Availability

The raw proteomic data obtained from SWATH-MS analysis of total exosomes and placenta-derived exosomes have been provided as supplementary files. Specifically, the datasets for total exosomes and placenta-derived exosomes are available in Supplementary File 15 and Supplementary File 16, respectively.

## Supplementary material

Supplementary Figure S1 Screening of different groups of differentially expressed proteins and co-differentially expressed proteins in T-EXO and P-EXO A.PCA plot among QC, SICP, MICP and CTR group in T-EXO. B. PLS-DA validation plot among QC, SICP and MICP group in T-EXO. C. PLS-DA validation plot (permutation = 200) in T-EXO. D-G. Volcano plot of DEPs in T-EXO, the number of up-regulated proteins and down-regulated proteins. H.PCA plot among QC, SICP, MICP and CTR group in P-EXO. I. PLS-DA validation plot among QC, SICP and MICP group in P-EXO. J. PLS-DA validation plot (permutation = 200) in T-EXO. K-N. Volcano plot of DEPs in T-EXO, the number of up-regulated proteins and down-regulated proteins.

Supplementary Figure S2 Screening and bioinformatics analysis of common differentially expressed proteins in different groups of total and placental-derived exosomes A. VENN maps of the three groups of differential proteins in T-EXO. B. GO analysis of common DEPs in T-EXO. C. VENN maps of the three groups of differential proteins in P-EXO. D. GO analysis of common DEPs in P-EXO (PDF).
