# Supplementary figures and images for: Analysis of plasma exosomal differential proteins and bioinformatics in intrahepatic cholestasis of pregnancy
Source: Front Glob Womens Health. 2026 Apr 10;7:1751936. doi: 10.3389/fgwh.2026.1751936 (PMC13106368; doi:10.3389/fgwh.2026.1751936)

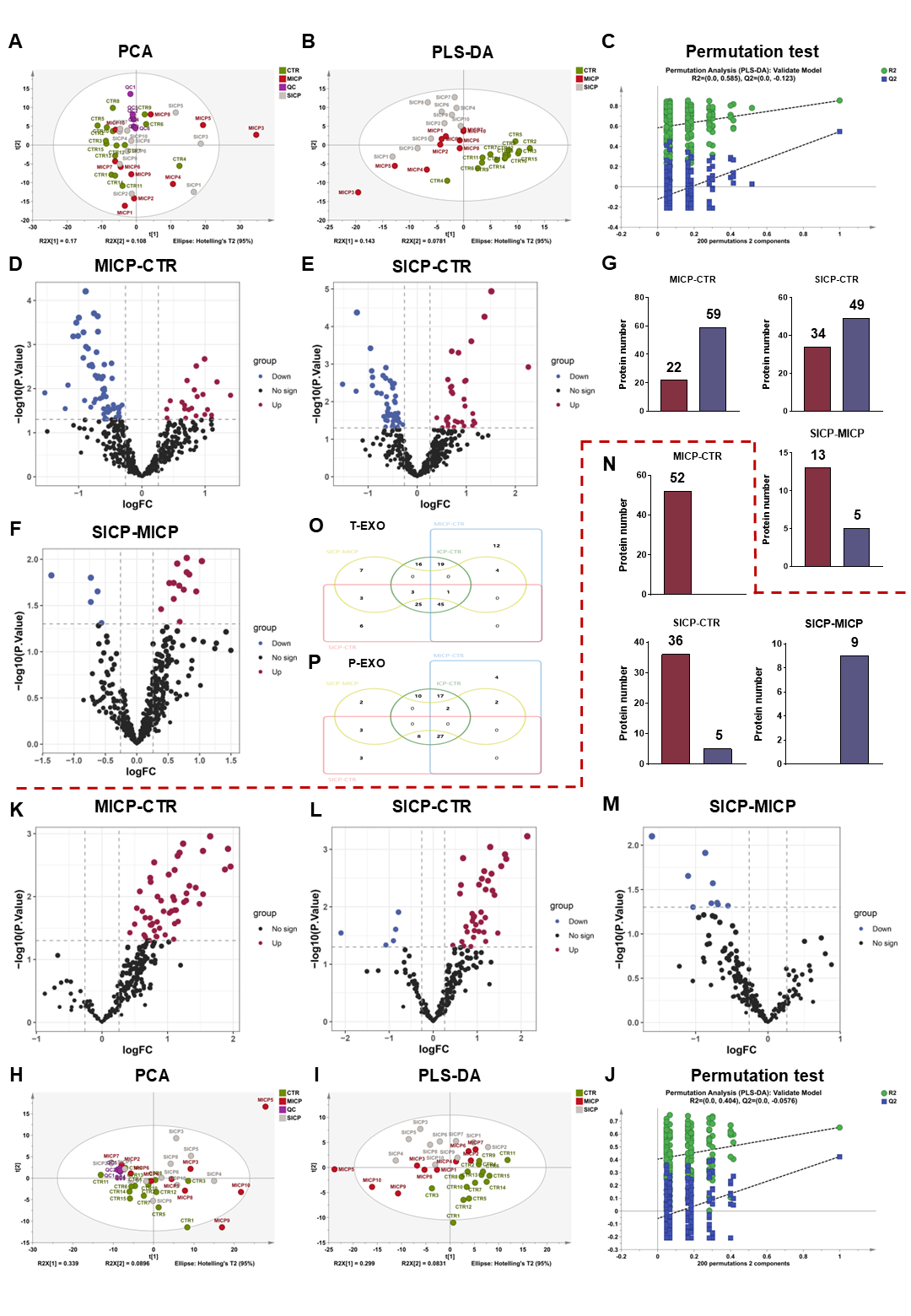

Supplement: Supplementary Figure S1 — Screening of different groups of differentially expressed proteins and co-differentially expressed proteins in T-EXO and P-EXO A.PCA plot among QC, SICP, MICP and CTR group in T-EXO. B. PLS-DA validation plot among QC, SICP and MICP group in T-EXO. C. PLS-DA validation plot (permutation = 200) in T-EXO. D-G. Volcano plot of DEPs in T-EXO, the number of up-regulated proteins and down-regulated proteins. H.PCA plot among QC, SICP, MICP and CTR group in P-EXO. I. PLS-DA validation plot among QC, SICP and MICP group in P-EXO. J. PLS-DA validation plot (permutation = 200) in T-EXO. K-N. Volcano plot of DEPs in T-EXO, the number of up-regulated proteins and down-regulated proteins. [file Image1.png]

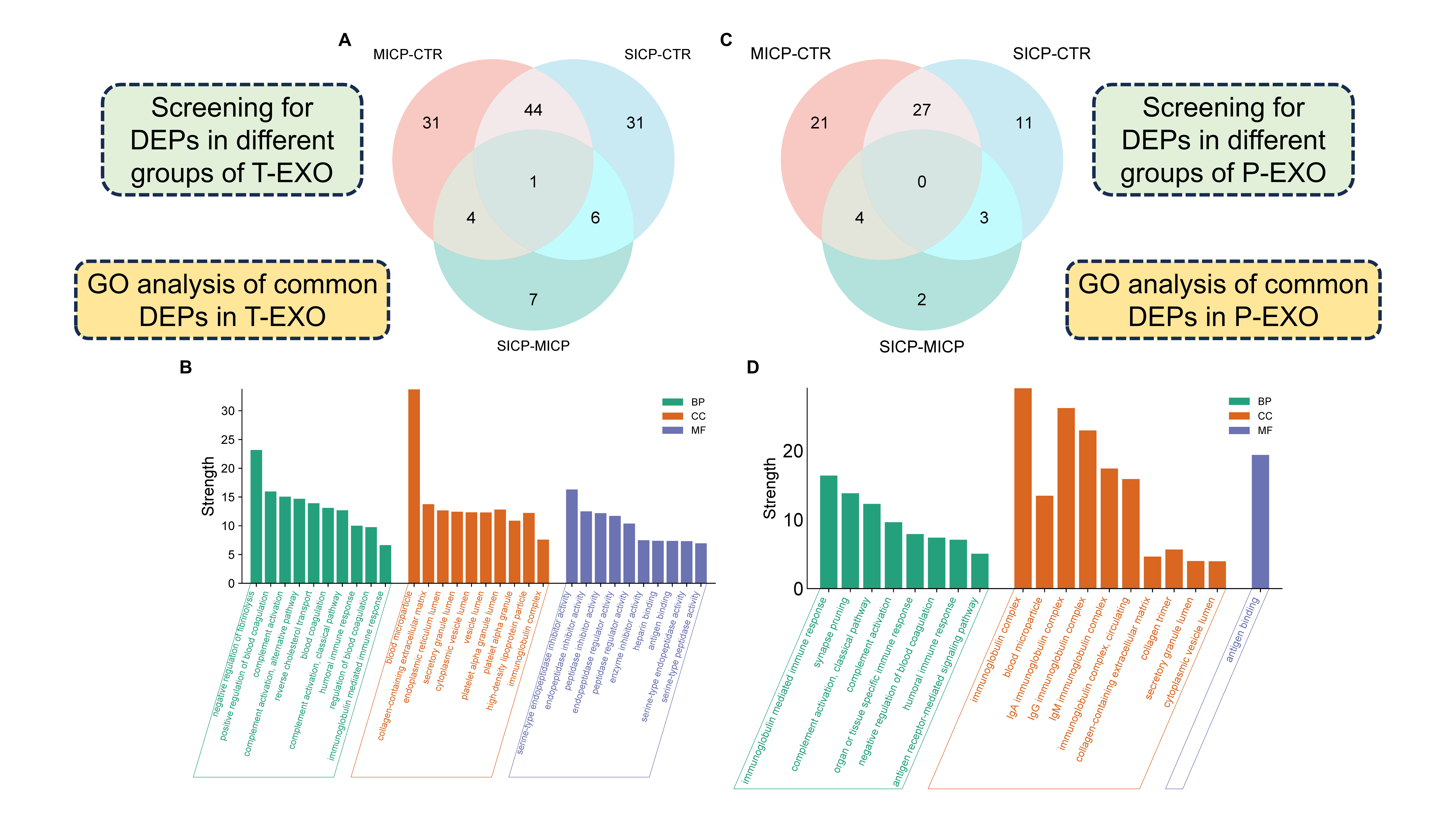

Supplement: Supplementary Figure S2 — Screening and bioinformatics analysis of common differentially expressed proteins in different groups of total and placental-derived exosomes A. VENN maps of the three groups of differential proteins in T-EXO. B. GO analysis of common DEPs in T-EXO. C. VENN maps of the three groups of differential proteins in P-EXO. D. GO analysis of common DEPs in P-EXO (PDF). [file Image2.tif]
